# Social media and internet use among orthopedic patients in Germany—a multicenter survey

**DOI:** 10.3389/fdgth.2025.1486296

**Published:** 2025-04-14

**Authors:** Yasmin Youssef, Tu-Lan Vu-Han, Richard Trauth, Georg Osterhoff, David Alexander Back, Tobias Gehlen

**Affiliations:** ^1^ Department of Orthopedics, Trauma and Reconstructive Surgery, University Hospital Leipzig, Leipzig, Germany; ^2^ Center for Musculoskeletal Surgery, Charité—Universitätsmedizin Berlin, Corporate Member of Freie Universität Berlin and Humboldt-Universität zu Berlin, Berlin, Germany; ^3^ Berlin Institute of Health at Charité—Universitätsmedizin Berlin, BIH Biomedical Innovation Academy, Berlin, Germany; ^4^ Hessing Klinik, Augsburg, Germany; ^5^ Move Ahead—Foot, Ankle and Sportsclinic, Berlin, Germany

**Keywords:** social media, digitalization, patients, communication, orthopedics

## Abstract

**Background:**

Social media (SM) is increasingly used in the healthcare system and offers various benefits for patients such as accessible health information and communication with other patients and healthcare professionals. However, SM also poses risks, including the dissemination of medical misinformation and privacy concerns. This in turn can influence patients’ health-related decision-making and the patient-physician relationship. There is limited data regarding which SM orthopedic patients use and what benefits and risks of SM they perceive.

**Methods:**

An online survey was conducted from April to December 2023 among orthopedic and trauma patients in five German orthopedic clinics. The questionnaire with 32 variables was designed to assess internet and SM usage patterns, platform preferences, and perceived benefits and risks. Statistical analysis was performed, including subgroup analyses.

**Results:**

A total of 267 patients participated, with 82.0% reporting regular SM use. In total 45.9% of the patients used SM for general health questions and 51.3% for orthopedic-related questions. The most used information platforms were conventional websites, YouTube, Instagram, and messenger apps. A total of 45.9% used SM infrequently for general health questions, and 51.3% for orthopedic-related queries. Only 13.7% of patients agreed that SM helped in medical decision-making, and 31.1% felt confident in assessing the credibility of SM content. Additionally, 58.6% of patients were unsure about allowing physicians to present their cases on SM, and 62.3% were uncertain about posting their medical images.

**Conclusion:**

Among German orthopedic patients, the use of SM for health-related and gain of orthopedic information was low in the given study. While SM may offer valuable health information, their role in medical decision-making remains limited due to concerns over content credibility and privacy. Video-based content seems to achieve the best reach. Future research should explore these aspects longitudinally and across diverse populations to better understand and address the challenges and benefits of SM in healthcare.

## Introduction

The rapid expansion of social media (SM) has transformed and revolutionized the communication landscape by connecting individuals across the globe and providing unprecedented opportunities for information dissemination and social interaction (1–4). SM influence almost all aspects and areas of daily life and are also increasingly used as a means for communication in the healthcare system, used by patients, healthcare professionals, and healthcare-providing institutions alike (4, 5).

SM use among patients may bring various benefits, such as accessibility to health information, exchanging personal experiences, and emotional support (4–6). These benefits have been particularly noted for patients with chronic conditions such as diabetes and heart disease (7–10). However, there are also potential risks and caveats associated with SM used in the healthcare system like the dissemination of misinformation and privacy and medico-legal concerns (11–13). Misinformation on social media can influence patient decision-making, potentially leading to delays in seeking appropriate care, adherence to ineffective or even harmful treatments, and increased distrust in medical professionals. This is particularly concerning in the context of evidence-based medicine, where treatment decisions should be guided by the best available clinical research. When patients rely on unverified online sources rather than established medical guidelines, it can compromise optimal recovery, long-term health outcomes, and overall trust in the healthcare system. The use of SM by patients also influence the dynamics of the relationship between patients and healthcare professionals (6, 14). However, it must also be critically noted that while it can lead to more informed, equal, and harmonious communication between patients and healthcare professionals, it can also contribute to a higher rate of doctor-switching and suboptimal interactions between patients and healthcare professionals (6). While this may offer broader perspectives, it also raises concerns about potential fragmentation of care, particularly in orthopedic practice, where continuity, long-term treatment planning, and follow-up care are crucial for optimal outcomes.

While current studies have discussed the use of SM by orthopedic and trauma surgeons, the literature on patients’ use of SM in the field of orthopedic and trauma surgery remains limited (15–18). Acknowledging the prominent role of SM in daily life and their possible impact on the dissemination of healthcare information, it becomes imperative to explore and understand the implications of SM as well as the consumption and usage behavior of patients also in the field of orthopedics.

The aim of this study was to gain current insights on general health-related internet use and the use of SM among German orthopedic and trauma patients, regarding the platforms used, their usage behavior and their perceived benefits and risks.

## Methods

### Study design

An online questionnaire was designed to assess the current health-related internet use and SM use among orthopedic patients in Germany and was administered using SurveyMonkey (SurveyMonkey, Palo Alto, CA, USA). The questionnaire was distributed to orthopedic patients in five clinics in Germany (three university hospitals, one maximum-care hospital, and one specialized clinic). Posters with QR codes were placed in the waiting rooms of the orthopedic and trauma surgery outpatient centers. This method was preferred over paper questionnaires due to its efficiency in data collection, ease of distribution, and reduction of logistical effort. The survey was conducted between April 2023 to December 2023.

### Ethical considerations

The data was processed in accordance with the European data protection regulations (19). This study adheres to the General Data Protection Regulation (GDPR) guidelines for the use of personal data. All participating patients received written patient information, explaining the aim and scope of the study as well as how the data would be collected, processed, and analyzed. Participation was voluntary. Data were anonymized prior to analysis to ensure privacy and confidentiality. While demographic information such as sex, age, and city size were included in the dataset, these variables were handled in a way that minimizes the risk of re-identification.

### Questionnaire design

The questionnaire was developed in two steps. In the first step, current literature on the topic of social media use by patients was reviewed by the authors and key areas of interest were identified. Subsequently, questions were formulated. In the second step, the preliminary questionnaire was tested on three patients. The questionnaire was then finalized considering the feedback on content, practicability, and understandability from the pilot group. The final questionnaire consisted of 32 variables containing demographic questions, questions on internet and SM usage behavior, specific health-related uses of SM, digital literacy and the perceived advantages of SM for medical purposes. Specific uses of different SM platforms that were asked for were: receiving information on the medical condition, treatment options, side effects of medication, prevention methods and possibilities, clinics and outpatient practices and communication with other patients, referring to other patients experience and sharing of own experience. A 5-point-Likert scale (from 1 = “I strongly disagree” to 5 = “I strongly agree”) was used to assess digital literacy and the perceived advantages.

### Data processing and statistical analysis

Data processing, visualization, and descriptive statistics were performed using Python 3 (version 3.11) (20). The statistical analyses were performed using SPSS (IBM SPSS Statistics® for Mac, version 29.0.1.1, Chicago, Illinois, USA). Categorial data was presented in frequencies (n) and percentages (%). Because not all respondents answered every question, denominators differ among survey items. Subgroup analysis was performed for sex (male vs. female) and age. For the analysis of age, the data was grouped into five different generations: ages 0–13 (generation alpha), ages 14–27 (generation Z), ages 28–43 (generation Y), ages 44–59 (generation X), and ages >60 (boomers). The chi-squared test was used for categorical data to assess differences between groups. The level of statistical significance was set at a two-sided *P*-value of <0.05.

## Results

### Demographics of survey participants

A total of 267 orthopedic patients participated in the online survey. In total 47.2% (126/267) identified as males, 52.4% (140/267) as females, and 0.4% (1/267) as diverse. The mean age of participants was 43.81 years (SD 17.13), with a median of 46.0 years. Of the patients, 46.1% (123/267) lived in a large city (> 100,000 inhabitants), 24.7% (66/267) lived in a rural community (< 5,000 inhabitants), 15.7% (42/267) lived in a small-sized town (<20,000 inhabitants), and 13.5% (36/267) in a medium-sized town (20,000–100,000 inhabitants). Within the patient cohort, 28.8% (77/267) presented to an orthopedic or trauma surgeon because of an acute traumatic injury, 28.1% (75/267) for a chronic, degenerative condition, 16.9% (45/267) for sports injuries, and 12.7% (34/267) for a postoperative follow-up, 7.1% (19/267) discussion of results, or 6.4% (17/267) other.

### SM usage among orthopedic patients

**Figure 1:**
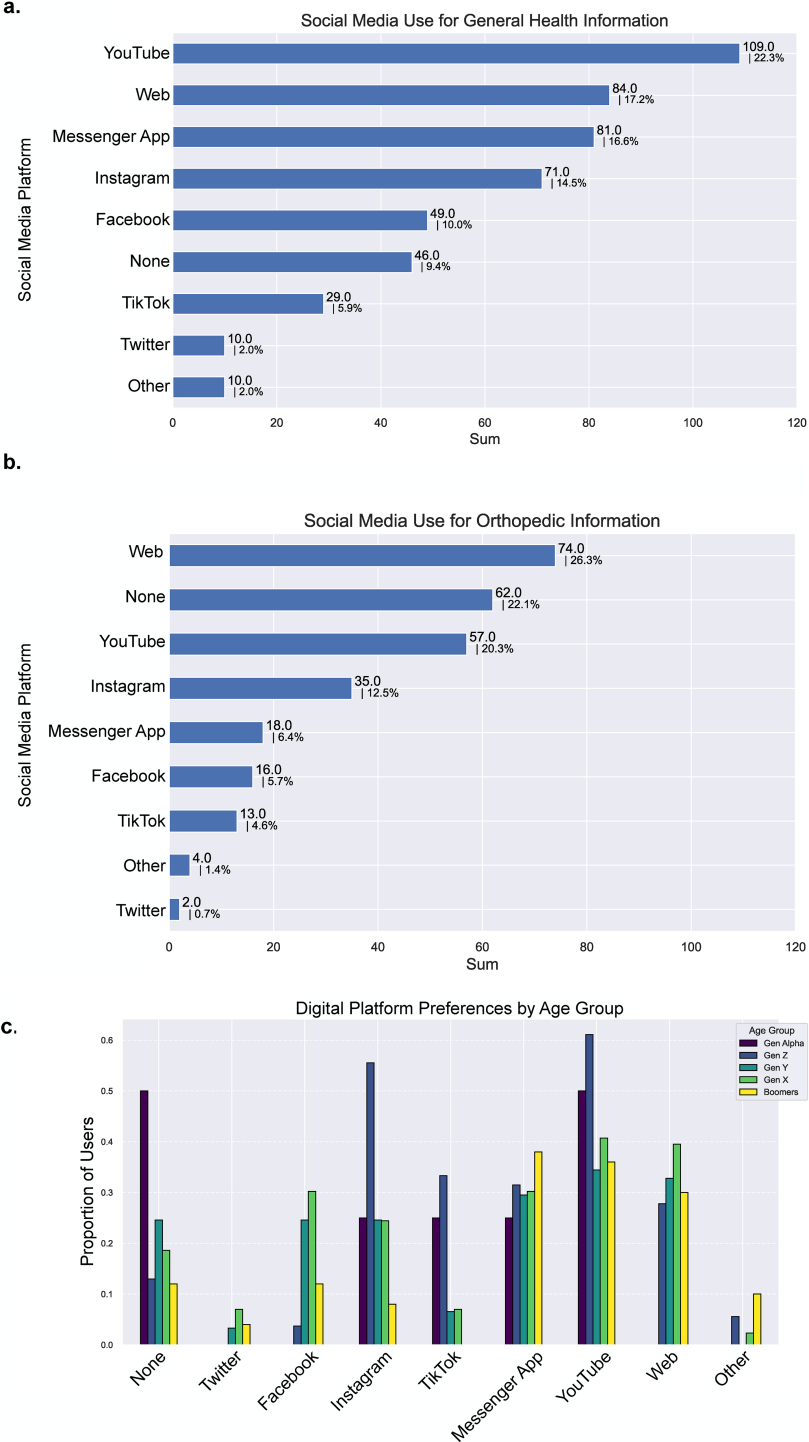
**(a)** social media use for general health information ranked by frequency (255 responses) **(b)** social media use regarding orthopedic and trauma questions ranked by frequency (197 responses) **(c)** digital platform preferences according to age group.

Of the survey participants 82.0% (209/255) stated that they use SM. Of the patients, 45.9% (117/255) and 51.3% (101/197) reported infrequent use of social media for general health and orthopedic-related questions respectively. Further, 16.1% (41/255) stated that they never use SM for general health questions and 26.9% (53/197) stated that they never use SM for orthopedic-related questions. The most frequently used SM platforms for general health information were YouTube (22.3%; 109/489), followed by conventional websites (17.2%; 84/489), and messenger platforms (16.6%; 81/489) (Figure 1a). The platforms used most frequently regarding orthopedic and trauma information were the conventional websites (e.g., hospital websites, health authority websites, standard online publications) (26.3%; 74/281), followed by YouTube (20.3%; 57/281), and Instagram (12.5%; 35/281) (Figure 1b). Here, it should be noted that (22.1%; 62/281) stated that they use no SM platform for orthopedic and trauma purposes compared to (9.4%; 46/489) for general health information. Figure 1 presents the SM platforms used for general health (Figure 1a) and orthopedic questions (Figure 1b).

### Patterns of digital platform preferences by age group

A subgroup analysis by age brackets highlighted preferences for Instagram and Youtube among ages 14–27 (generation Z), whereas there was a preference for YouTube and web platforms among ages 28–43 (generation Y), and 44–59 (generation X), respectively. Engagement with digital platforms among ages 0–13 (generation alpha) was relatively low (None) and focused primarily on YouTube, Instagram, TikTok, and messenger apps (Figure 1c).

### SM usage for the acquisition of specific health-related questions

**Figure 2:**
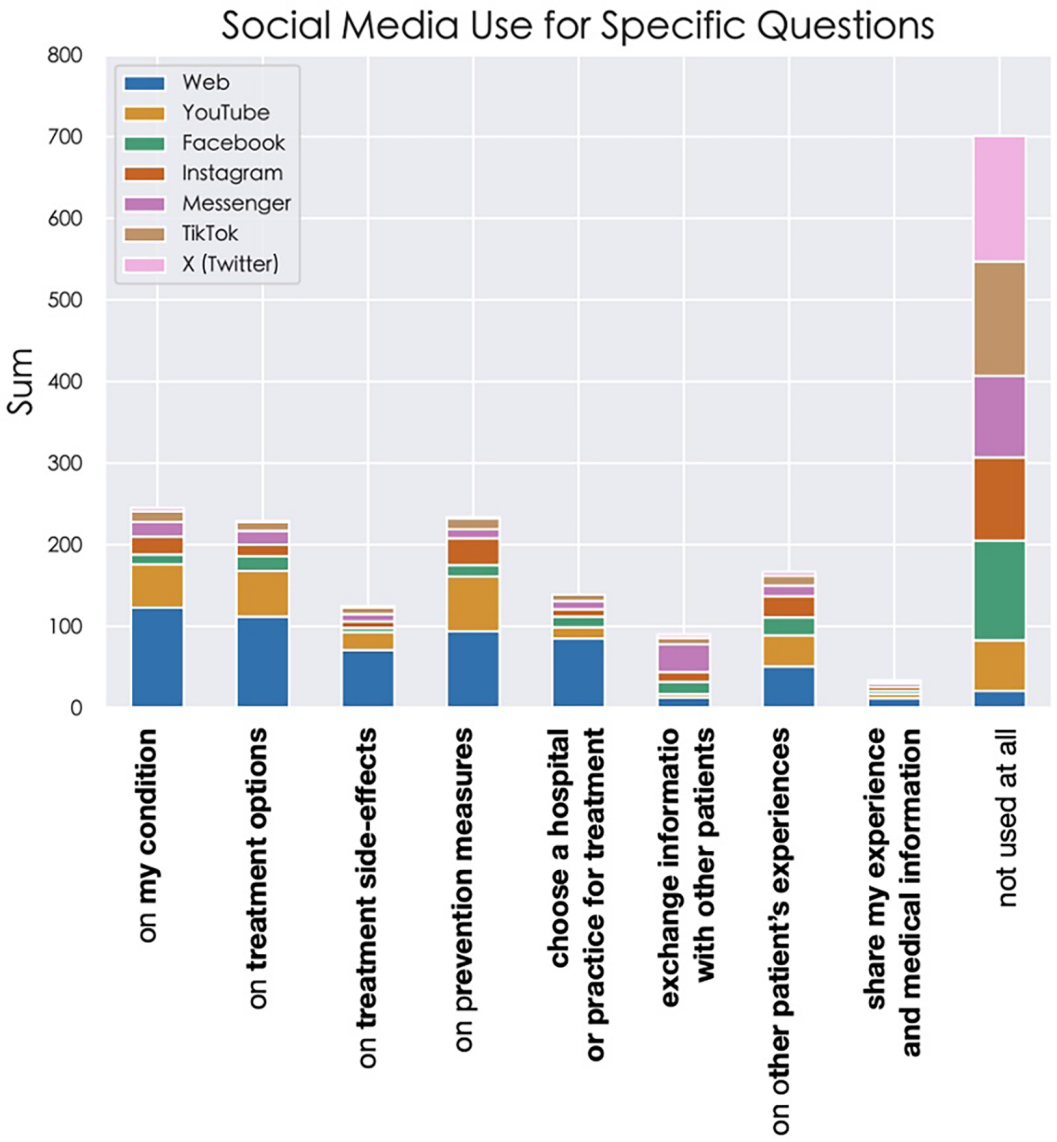
Specific health-related uses of social media by platform.

The highest specific uses were recorded for YouTube and Instagram, while the lowest was recorded for X (formerly Twitter) and TikTok. YouTube was the platform used most often by the patients for receiving information on their medical condition diagnosis (53/331; 16.5%), treatment options (56/331); 17.4%), medication side effects (22/331; 6.9%), and prevention possibilities (67/331; 20.9%). Messenger Platforms were used the most for communication with other patients (34/216; 15.7%). Information on other patient's experiences was received most frequently on YouTube (38/321; 11.8%) and Instagram (26/231; 11.3%). Conventional websites were used more than any other social media platform for most of the specific uses, except for receiving other patients’ experience and finding information on a clinic or outpatient practice. More patients also used YouTube, than conventional websites for obtaining information on prevention methods and possibilities. A full analysis of the specific uses of SM and conventional websites for health-related questions is presented in Figure 2.

### Use of SM for health-related decision-making

**Figure 3:**
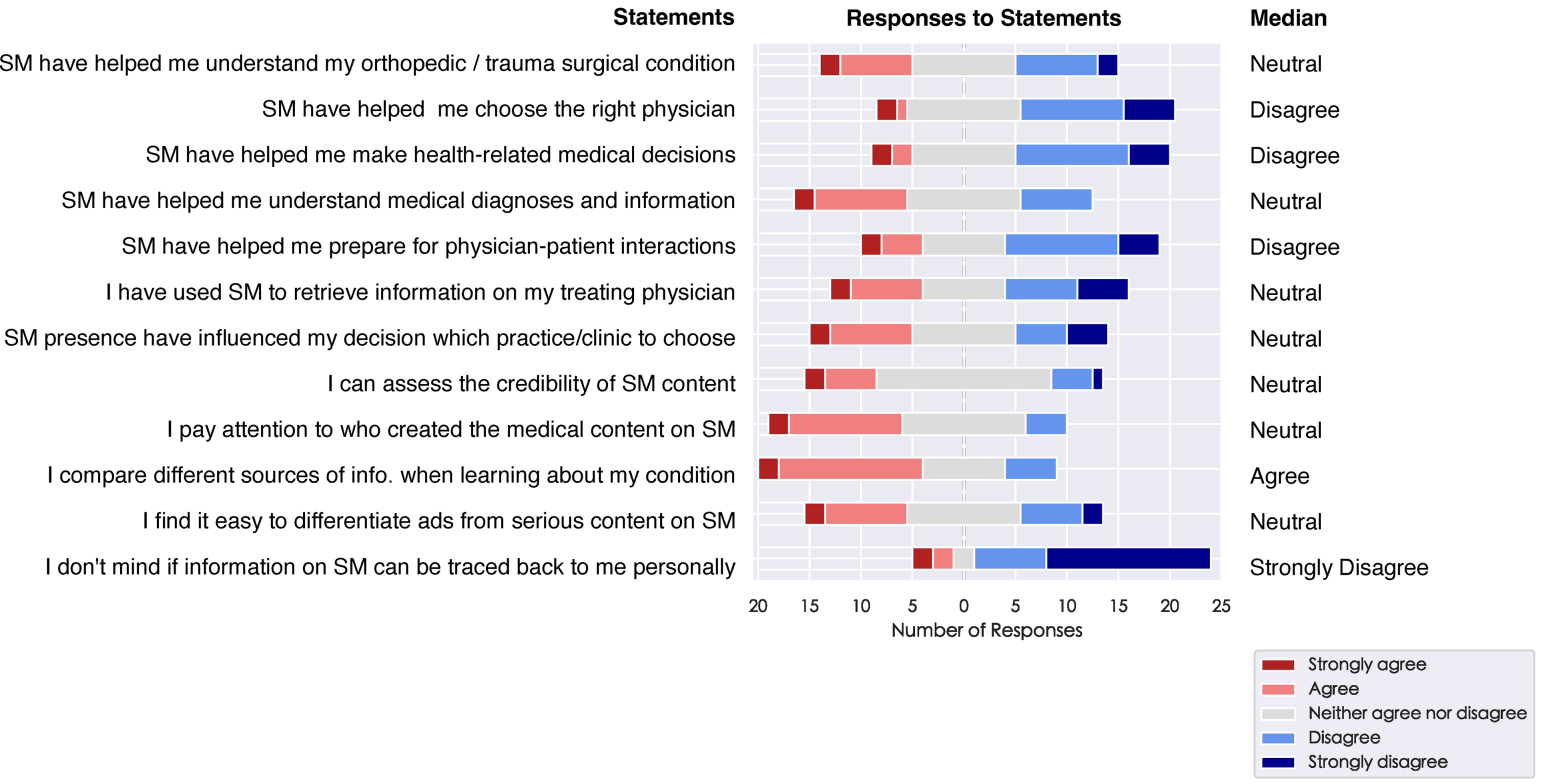
Plotting of Likert-scale responses to statements on health-related decision-making and self-stated SM competences.

In total 55.6% (65/117) of the patients disagree or strongly disagree that SM has helped them make medical decisions, while only 13.7% (16/117) agree or strongly agree. Likewise, most of the participants (59.8%; 70/117) disagree or strongly disagree that they have used SM for the preparation of patient-physician interaction. Of the study participants more than one third (38.8%; 50/129) stated that they would rate the statement that SM has helped them in understanding medical diagnosis and information as neutral, while 24.0% (31/129) agreed or strongly agreed and 37.2% (48/129) disagreed or strongly disagreed. 40.2% (51/127) of the participants have stated that they disagree or strongly disagree that SM has helped them to understand their orthopedic trauma surgical condition, while only 31.5% (40/127) strongly agreed or agreed to that statement. More than half of the participants (57.9%; 66/114) revealed that they strongly disagree or disagree that SM has helped them in choosing a physician, and 12.8% (14/114) agreed or strongly agreed. In total 34.3% (34/99) stated that they have already retrieved information from their treating physician on SM and 26.2% (26/99) stated that SM presence has already influenced their choice in a clinic or outpatient practice. Patients felt strongly about information being traced back to them personally. All statements and the median Likert-Scale responses are presented in Figure 3.

### Survey participants report low SM competence

Half of the participants (50.3%; 76/151) stated that they were not sure if they could adequately assess the credibility of SM content. Only 31.1% (47/151) stated that they can assess SM credibility. Most of the participants (52.7%; 78/148) stated that they pay attention to who created SM content (i.e., a medical professional). 15.5% (23/148) disagreed or strongly disagreed and 31.8% (47/148) were undecided when asked whether they pay attention to SM creators. Similarly, 50.0% (72/144) of the participants stated that they compare different sources of information when consuming medical information. However, in total 34.7% (50/144) stated that they were uncertain about this statement. In addition to that, 43.0% (65/151) were unsure if they could differentiate between serious SM content and commercial content, while only 32.5% agreed or strongly agreed that they had the competence to differentiate misinformation.

Figure 3 summarizes the median Likert-Scale values of the participants’ self-stated SM competences.

### Readiness for physicians posting patient content on SM

Most patients (58.6%; 89/152) were unsure whether they would allow their physician to present their case on SM. Overall, 35.5% (54/152) of respondents indicated that they would allow their case to be presented on social media. Among them, 68.5% (37/54) stated that they would not object if their physician were to share their case without explicitly seeking their permission, though in practice, obtaining patient consent remains essential. Similarly, most of the patients (62.3%; 94/151) were unsure whether they would allow their physician to post their images (i.e., x-rays, CT scans, MRIs) on SM. 30.5% (46/151) would allow their images to be posted, of which 69.6% (32/46) would allow this without their physician to ask for permission.

### Correlation studies

SM literacy and navigating different platforms may depend on age and sex. To test, whether we could observe a sex-dependent use of SM, we performed a subgroup analysis and a two-proportion z-test and found no significant difference between males and females (*p*-value = 0.243), diverse patients were underrepresented in the data set (*n* = 1). We observed a similar use of SM by age group with generations Z, Y, and X reporting SM use in 23.6%, 27.9%, and 32.2%, respectively. To compare the different age groups, we performed a chi-square test, which showed no statistically significant difference between the different age groups (*p*-value=1).

## Discussion

This study provides an insight into social media (SM) and general internet use among orthopedic patients in Germany, highlighting their usage patterns, platform preferences, and perceived benefits and risks.

With 82%, most of the participating patients stated that they used SM, in line with previous publications that reported that over 76% of patients relied on social media at least in some way concerning health-related concerns (21). Up to 80% of cancer patients use digital platforms to connect and exchange information (5), and 85% of Saudi patients sought health information using social media (22). However, the use for SM platforms seemed to be limited to general health-related purposes and for the orthopedic-related queries in this sample population. Approximately half of the patients indicated that they used SM infrequently for health-related or orthopedic-related questions. However, a relevant number of the patients also noted no use of SM for those purposes. The most used platforms were conventional websites, YouTube, Instagram, and messenger apps. Conventional websites were also listed, since such sources of medical information were preferred to SM in the presented patient cohort. Interestingly, messenger apps, YouTube and conventional websites have also been shown to be the most used platforms for the professional use of SM among German orthopedic and trauma surgeons, suggesting a potential regional preference (16). YouTube was the platform used most often by patients for receiving information on their medical conditions or diagnoses, treatment options, medication side effects, and prevention possibilities. Among orthopedic surgeons in Germany, the most used platforms for sharing health-related information were messenger apps, job-oriented SM (e.g., LinkedIn and ResearchGate), Facebook, and Instagram (15).

In addition, it was shown that SM played a subordinate role in health-related decision-making. In the cohort presented, more than half of the respondents stated that SM has not helped them in making medical decisions. Similarly, more than a third of the respondents stated that SM had not helped them to better understand their medical diagnosis. These findings might indicate a general skepticism or cautious engagement with SM for medical purposes among patients. On the other hand, this might also be due to the limited SM content available in the area. Most online health-related resources and SM content are predominantly available in English (e.g., PubMed, WebMD, MedlinePlus, Mayo Clinic), with significantly fewer materials accessible in German. This disparity may pose a challenge for German-speaking individuals seeking health information, as understanding complex medical content in a non-native language can be difficult and render such information inaccessible. The introduction of generative AI may change this, although the usage and reliability of these upcoming resources have not been tested extensively. In two recent studies that analyzed the professional SM use of German orthopedic and trauma surgeons, it was suggested that SM might not yet be used to its full potential (15, 16).

Only a minority of the study participants stated to use SM for the acquisition and communication with patients and for sharing health-related information and clinical expertise (15). In addition to that, only a minority stated that they produce their own content on SM (16). In the presented study, a total of 34.3% of the patients indicated that they had already retrieved information from their treating physician on SM and 26.2% stated that SM presence had already influenced their choice of a clinic or outpatient practice. Only 12.8% agreed that SM has already helped them choose a physician. These findings are similar to those presented by Johnson et al., where 19% of the respondents stated that they were likely to view the SM account of their physician and 23% felt that SM is likely to influence the physician they see (23).

Despite these benefits, the study results also highlight significant risks associated with SM use in healthcare. A substantial proportion of the patients lacked confidence in assessing the credibility of SM content, with 50.3% being unsure, if they can adequately evaluate the trustworthiness of information encountered on SM. These results indicate a need for improved digital and medical literacy among patients as well as improvement of detection of misinformation in digital platforms. To address this, it is crucial to provide patients with strategies for identifying reliable sources of information. Patients should be encouraged to look for content from reputable institutions such as government health agencies, university hospitals, and professional medical associations. Furthermore, verifying whether health information is supported by scientific studies or expert consensus can enhance reliability. In a current study, Song et al. showed that there are considerable differences in the habits and patterns of online health information seeking and the perceptions of online health information sources (24). In addition to that, it was shown that adolescents show trends towards troubling digital health literacy and their trust in those sources is dependent on multiple factors such as trust in other users and content (25). In a study, it was shown that adolescents would like to enhance their digital health literacy to be able to appraise the health information that they encounter online. They also stated that they seldom discussed the health information that they found online with health professionals, but they would appreciate the opportunity to have such discussions and learn how to locate and evaluate online health information (26). The dissemination of misinformation is a major concern, as incorrect or misleading health information can lead to poor health outcomes (11–13). Studies have shown that more than half of the health-related videos on digital platforms contain misleading information or biases (27). Certain platforms may offer higher-quality content, such as verified channels on YouTube, fact-checked medical articles on professional websites, and social media pages managed by healthcare institutions. Encouraging patients to utilize these sources can contribute to more informed healthcare decisions. This gap in digital literacy points to a critical area where healthcare providers can intervene, offering guidance on how to navigate and evaluate online health information effectively. Moreover, healthcare professionals should consider leveraging social media to educate and inform patients by sharing verified, easy-to-understand medical content, addressing common misconceptions, and fostering open discussions through interactive formats such as Q&A sessions or live streams. Moreover, it is important to recognize different patient segments with distinct health literacy and SM behaviors. Tailoring communication strategies to these different patient groups can enhance the effectiveness of digital health interventions. Practical ways to incorporate SM into routine patient education include curated lists of credible resources.

Privacy and medico-legal concerns also emerged as critical issues. The willingness of patients to allow their physicians to post their cases or medical images on SM was low. This reluctance reflects apprehensions about confidentiality and data security, emphasizing the need for stringent privacy protections and clear guidelines for healthcare professionals when engaging with patients on SM. It had become a common practice for health professionals to share patient cases, including patient data on SM for teaching and sharing medical expertise (28, 29). The medical history and images that are shared may be identifiable (28). This poses a significant risk to individual patient privacy and data protection regulation, which are both fundamental rights within the European Union and the General Data Protection Regulation (19).

This study is subject to certain limitations, primarily related to its sample and methodology. First, surveys have minor levels of evidence in general. In addition, the survey was conducted in Germany as specific geographic region and relied on self-reported data, which may introduce a bias, as the outcome can be affected by the participants’ understanding of the questions. Using an online questionnaire may have also influenced the representativeness of the study population, as individuals with lower digital literacy or limited access to online platforms might have been less likely to participate, potentially introducing a selection bias. Furthermore, the participants’ population of 267 patients can hardly be considered as being representative of all orthopedic and trauma patients in Germany. Finally, due to the voluntariness of participation, patients with a higher affinity towards SM use might be overrepresented, posing another potential bias. The presented results must therefore be treated with caution. Additionally, the cross-sectional design captures SM use at a single point in time, precluding analysis of trends or changes over time. Furthermore, qualitative research, for example in the form of qualitative interviews, to capture why patients remain uncertain or distrustful about social media health content would be of great interest.

Overall, this study has added new insights to the current literature on the SM usage behavior of patients and their physicians, especially in the field of orthopedics. The results indicate that the participating patients did not regularly use SM for retrieving health-related information, that they assign SM a subordinate role in health-related decision-making, that they were insecure using SM for medical purposes and expressed concerns when it comes to physicians presenting patients data online.

Future research should aim to address these limitations by incorporating longitudinal designs and expanding the geographic scope to include diverse populations. Moreover, investigating the effectiveness of interventions designed to improve SM literacy among patients could provide valuable insights into mitigating the risks associated with SM use. Understanding how different demographic groups utilize SM can also inform targeted educational campaigns and support services tailored to varying needs and competencies.

## Conclusion

This study highlights that while most orthopedic and trauma patients in Germany use social media, its role in the acquisition of health data in the field of orthopedics and trauma surgery and in health-related decision-making is limited. Many patients expressed skepticism and concerns about credibility and privacy on SM. These findings indicate a need for improved digital health literacy and better utilization of social media by healthcare professionals. Healthcare professionals should use social media to share verified medical content, correct misconceptions, and engage with patients in a professional way. This study is limited by its survey-based, self-reported data from a specific German population and a cross-sectional design that prevents trend analysis. Future research should address these issues and explore interventions to enhance patients’ confidence and competence in using social media for health purposes.

## Data Availability

The raw data supporting the conclusions of this article will be made available by the authors, without undue reservation.
